# The Grand Challenge – Managing End-Staged Joint Osteoarthritis

**DOI:** 10.3389/fsurg.2014.00009

**Published:** 2014-04-22

**Authors:** Peter F. Choong, Michelle M. Dowsey

**Affiliations:** ^1^University of Melbourne, Department of Surgery, St. Vincent’s Hospital Melbourne, Melbourne, VIC, Australia; ^2^Department of Orthopaedics, St. Vincent’s Hospital Melbourne, Melbourne, VIC, Australia

**Keywords:** osteoarthritis, joint replacement, patient selection, sustainability, registries

## What is the Problem?

Musculoskeletal disease is a common condition that affects up to one-quarter of the population over 65 years in many developed countries. Over 6 million Australians (1) and 27 million Americans (2) are affected by some type of musculoskeletal disorder of which osteoarthritis (OA) is the most common. Musculoskeletal disorders account for one-fifth of all consultations with general practitioners (3) in Australia. The total treatment cost of OA and other musculoskeletal conditions in Australia was estimated as $55.1 billion in 2012, and the burden of disease cost was estimated to be $34.2 billion, based on a loss of 182,135 disability adjusted life years (DALYs) (4). In the United States, the personal and community cost of musculoskeletal diseases including those through lost wages now approaches $950 billion (5). The 2010 Global Burden of Disease Study demonstrates the worldwide scale of this problem, identifying musculoskeletal disorders as the second largest contributor to life lost through disability after Cancer (6). OA has the highest trajectory of increasing prevalence across all musculoskeletal conditions (4) and in Australia is forecast to become the leading cause of disability (7). Total joint replacement (TJR), which is not only effective for improving the quality of life of people with end-stage OA (8), is also a cost-effective solution (9).

## What is the Challenge?

What is required is a system-wide reform of the management of end-stage OA beginning with how patients with OA are assessed for referral to orthopedic surgeons for TJR, how the appropriateness for referral is decided, how patients who are being considered for TJR are re-evaluated while co-morbidities are addressed, and finally how patients who are not suitable for TJR are identified and referred for alternate care. The central theme that unites these processes and which is the essence of this grand challenge is improving outcomes after TJR by determining *Appropriate Patient Selection* – the right treatment for the right patient at the right time.

## What are the Dilemmas When Considering TJR?

Total joint replacement can be the treatment of choice for OA, but there is little evidence to guide decision-making about who is most likely to benefit from TJR, nor the best timing of when surgery should be performed (10). There are many factors complicating the decision-making process.

### High volume

Referral guidelines for TJR have been promulgated by many learned colleges and consumer advocacy groups (11). However, referrals for TJR are becoming so prolific that the numbers on surgical waiting lists may rise to unsustainable levels. For example, the almost 90,000 hip and knee TJRs performed in Australia (12) in 2011 is expected to double by 2020 (13). This mirrors international change (14) including the United States, where rises in hip (140%) and knee (670%) replacements over the next two decades will result in 570,000 hip and 3.5 million knee replacements being performed annually by 2030. The rate of increase of the utilization of TJR across all age groups is likely to exceed government projections and supply (15).

### High cost

While TJR for knee and hip OA is a cost-effective solution (9), the volume of procedures leads to a high cost burden for the public and private health sectors and is a major part of the health expenditure spent on treating OA annually (5, 16). A survey of insurer and out of pocket costs of OA in the United States found that these costs were not only substantial and may be prohibitive for those seeking definitive treatment of OA, but that the greater prevalence of women with OA and their more intensive use of health care accounted for two-thirds of the increase in health care expenditure resulting from OA (2).

### High risks

Although common, TJR is mainly performed on older patients with their attendant medical risks. A substantial rise in obesity is not only leading to more complicated outcomes of TJR surgery, but the prevalence is overrepresented (40–60%) in patients presenting for lower limb arthroplasty (17–19) increasing the risk for these high volume procedures.

### High dissatisfaction

Although the rate of revision surgery for failed TJR is only 0.5% per annum (20), making it to appear a highly successful procedure, 20–40% of patients remain dissatisfied. The main reason for this dissatisfaction is ongoing pain despite surgically satisfactory procedures. The rate of revision surgery alone may be an underestimate of failure of surgery (21) because dissatisfied patients continue to make ongoing demands for care from already overburdened healthcare resources despite not undergoing revision surgery.

### High variability in surgeon practice

Since the inception of the Swedish TJR registries for hip and knee OA, other registries now confirm the highly variable and at times paradoxical practices amongst surgeons. For example, the Australian Orthopaedic Association National Joint Replacement Registry has reported that totally cemented and hybrid total hip replacements consistently outperform total cementless replacements (20). Yet, the practice of implanting cementless prosthetic replacements continues to increase (20) with substantial cost implications because of the large cost differential between cemented and cementless prostheses. How surgeons use data and why pieces of evidence lack traction remains unclear.

## Important Issues in End-Stage OA Management

### Who will respond well to/benefit most from TJR?

Appropriate patient selection and response to TJR are intimately linked. Yet very little work has been done to clarify what is meant by appropriate patient selection or to understand what constitutes response to TJR. Recognizing who are poor responders, will allow clinicians to direct them to alternate non-surgical options for managing their OA or to undergo strategies to mitigate their risk profile prior to TJR so as to improve their response to surgery.

### Is the application of TJR equitable?

Variations in who receive TJR, and rising health care costs, have contributed to the need to ensure that the provision of TJR remains equitable, efficient, and safe in an environment of cost containment. The challenge for addressing the increasing demand for TJR is how to distribute limited resources, with the aim to preserve equity as part of the National systems of health care.

### How should TJR service be delivered in the future?

The inequity of insurance status’ and resources means that in some nations one sector absorbs the burden of TJR in place of another. For example, almost two-thirds of TJR in Australia are undertaken in the private health sector despite only one-third of Australians having private health insurance. It is not clear if the factors that drive good and poor response to TJR are the same in the public as in the private health arenas. Understanding the epidemiologic differences between patients from these two insurance categories will help future planning of service delivery and better inform models of care that are already in place.

### What is the impact of revision joint replacement?

Although prosthetic survival has improved significantly over the last two decades, the effect of rising numbers of primary TJRs is to increase the pool of patients who will require revision surgery in the future (22). The benefits of the current technology in enhancing fixation were not available before two decades or more and a surge in the requirement and expenditure for revision can be expected from TJR performed in the short term. It is anticipated that younger patients will comprise up to 50% of those requiring revision surgery (23).

## What is Required?

Improving the paradigm of care for people with OA is a bold and challenging task. What is required is to (i) build a world-class critical mass of expertise and resources harnessed from the best groups and institutions in the field, (ii) coordinate nationwide research to tackle the component parts of OA care, (iii) fertilize and link individual group research through broader collaboration, (iv) enhance musculoskeletal research through sharing and developing critical yet sparse skills sets such as health economics and agent-based research, (v) promote and facilitate multidisciplinary research approach through comprehensive stakeholder engagement, (vi) build a force of future researchers that will drive innovation and translation, (vii) bring together groups and resources to create opportunities for fundable research focused specifically at musculoskeletal health, and (viii) accrue data through the diligent use of joint replacement registries that will identify patterns of prosthetic performance that will inform safe, cost-effective, and sustainable strategies for TJR.

All this will not be possible by single institutions and informal collaborations alone. What is required is a concerted global effort by national peak bodies representing not only orthopedic surgeons but also other stakeholders to unite with funding and government agencies to develop context-specific strategies for addressing the demands, minimizing complications, improving outcomes and patient satisfaction, reducing costs, and increasing effectiveness and advantages, of primary and revision TJR. Creating the evidence to support all this is the grand challenge.

## Conflict of Interest Statement

The authors declare that the research was conducted in the absence of any commercial or financial relationships that could be construed as a potential conflict of interest.
